# Temporal Trends, Characteristics, and Citations of Retracted Articles in Cardiovascular Medicine

**DOI:** 10.1001/jamanetworkopen.2021.18263

**Published:** 2021-07-22

**Authors:** Raoul R. Wadhwa, Chandruganesh Rasendran, Zoran B. Popovic, Steven E. Nissen, Milind Y. Desai

**Affiliations:** 1 Cleveland Clinic Lerner College of Medicine, Case Western Reserve University, Cleveland, Ohio; 2 School of Medicine, Case Western Reserve University, Cleveland, Ohio; 3 Department of Cardiovascular Medicine, Heart, and Vascular Institute, Cleveland Clinic, Cleveland, Ohio

## Abstract

This systematic review examines temporal trends of retractions in cardiology literature and assesses their effect.

## Introduction

On certain occasions, the veracity of a published scientific report comes into question, resulting in its voluntary or involuntary retraction. This is a growing problem in modern medicine because the retraction rate of scientific articles has gradually but significantly risen over time.^1,2^ However, even postretraction, many articles continue to be cited, and nearly one-third of retracted articles are not labeled as retracted.^1^ This could have negative downstream ramifications, including wasted scientific resources because many articles whose findings are intricately tied to the retracted literature remain published with amplification of potentially false conclusions. Indeed, as of December 2020, 2 of the 10 most highly cited retracted publications pertain to cardiovascular medicine, and 19% of their combined citations (680 of 3499 citations) occurred postretraction.^3^ The temporal trends of retracted cardiology articles and their impact on downstream knowledge dissemination remain largely unexplored. This systematic review examines temporal trends of retractions in the cardiology literature and assesses their impact.

## Methods

This systematic review followed the Preferred Reporting Items for Systematic Reviews and Meta-analyses (PRISMA) reporting guideline. A comprehensive and high-quality database of 22 740 retracted publications was acquired from Retraction Watch.^3^ Articles in cardiology (445 [2%]) were programmatically extracted from the full database based on subject classification (Figure), and citation data were manually determined from unique digital object identifier–matched entries in Google Scholar. The study flow diagram is available in the eFigure in the Supplement. The Mann-Kendall trend test (S) was used for statistical inference (2-tailed, α = .05). The time-to-retraction trend was tested by setting a 5-year follow-up period to account for variable follow-up time. Statistical analyses were performed using R version 4.03 (R Project for Statistical Computing), and reproducible R scripts for all presented analyses are publicly available.^4^

**Figure.**
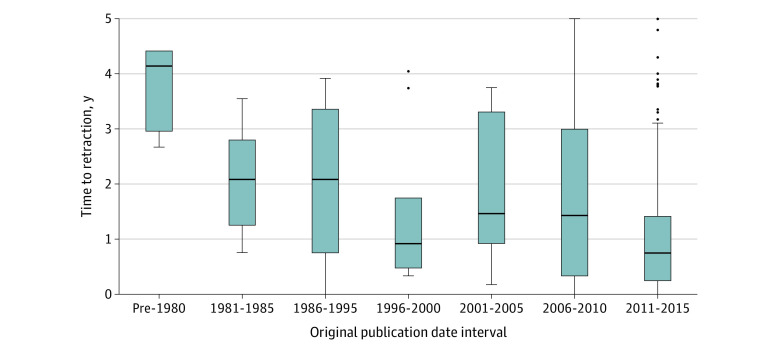
Effect of Article Publication Date on Time to Retraction Grouped boxplots of time between original publication and official retraction show a trend toward more rapid retractions for more recently published articles. Two 5-year intervals (ie, 1986-1990 and 1991-1995) were merged as each interval contained 2 data points after applying inclusion criteria, which was too few for analysis. The height of the box represents the interquartile range (IQR), the horizontal line in the box represents the median, the whiskers represent the 1.5 IQR of the 25th quartile or the 1.5 of the 75th quartile. The dots represent outliers.

## Results

A total of 22 740 retracted publications were acquired from the Retraction Watch database and included in this study, of which 445 (2%) were in cardiology.^3^ The most retraction-prolific author in cardiology contributed to 43 retracted articles (9.7%) (Table). In contrast, 1636 authors (85%) who contributed to a retracted cardiology article were authors with only a single retraction. In the 1990s, 1 to 2 of every 100 000 published cardiology articles were eventually retracted; by 2010, this number had more than doubled (Mann-Kendall: 20; S = 126; *z* = 4.1; *P* = .01). Additionally, 185 (42%) and 344 (77%) of cardiology retractions occurred within 1 and 5 years, respectively. Time to retraction was dependent on original publication date (Figure), with an overall downward trend indicating that, on average, recently published articles were retracted in a shorter time span than older articles (Mann-Kendall: 7; S = −15; *z* = −2.1; *P* = .04 for trend). The trend was unaffected by the removal of the most retraction-prolific author (*P* = .04 for trend). Approximately 420 retracted cardiology articles (96%) were cited fewer than 150 times, with 109 retracted cardiology articles (25%) between 25 and 150 times.

**Table.** Summary Statistics for Retractions in the Cardiovascular Medicine Literature and Medical Literature^a^

Description	Article, No
Retractions	445
Unique authors	1915
Retractions per author, mean (SD)	1.32 (1.47)
Retractions, median (IQR) per author	1 (1 to 1)
Maximum retractions per author, No./total No. (%)	43/445 (9.7)
Authors with multiple retractions, No./total No. (%)	279/1915 (14.6)

^a^ Summary statistics are reported as per author, accounting for authors who contributed to at least a single retracted article.

## Discussion

The number of retractions in the cardiology literature has increased sharply in recent years, even after adjusting for the increase in published articles. Several factors may have contributed to the increasing fraudulent retractions, including proliferation of predatory for-profit journals and the increasing competitiveness of academic environments, in which the number of published articles is often a proxy for success and promotion.^5^ This study was limited by its use of only a single database and the possibility that some studies that should be retracted are never identified. Although on average, retractions are being identified earlier, a small subset of impactful articles are cited disproportionately many times in the literature, even postretraction, and are thus responsible for large amounts of wasted resources spent by research based on their conclusions. Increased use of social media for research dissemination and critical analysis may be responsible for earlier identification.^6^ As a result, decreasing the number of retractions is an urgent and current issue that needs to be addressed within cardiology.

## Notes

Supplement. **eFigure.** Study Flow Diagram
