# Supplementary material for: Temporal Trends, Characteristics, and Citations of Retracted Articles in Cardiovascular Medicine
Source: JAMA Netw Open. 2021 Jul 22;4(7):e2118263. doi: 10.1001/jamanetworkopen.2021.18263 (PMC8299312; doi:10.1001/jamanetworkopen.2021.18263)
Supplement: Supplement. — eFigure. Study Flow Diagram [file jamanetwopen-e2118263-s001.pdf]

Supplemental Online Content

Wadhwa RR, Rasendran C, Popovic ZB, Nissen SE, Desai MY. Temporal trends, characteristics, and citations of retracted articles in cardiovascular medicine. *JAMA Netw Open.* 2021;4(7):e2118263. doi:10.1001/jamanetworkopen.2021.18263

eFigure. Study Flow Diagram

This supplemental material has been provided by the authors to give readers additional information about their work.

© 2021 Wadhwa RR et al. *JAMA Network Open*.

eFigure. Study Flow Diagram

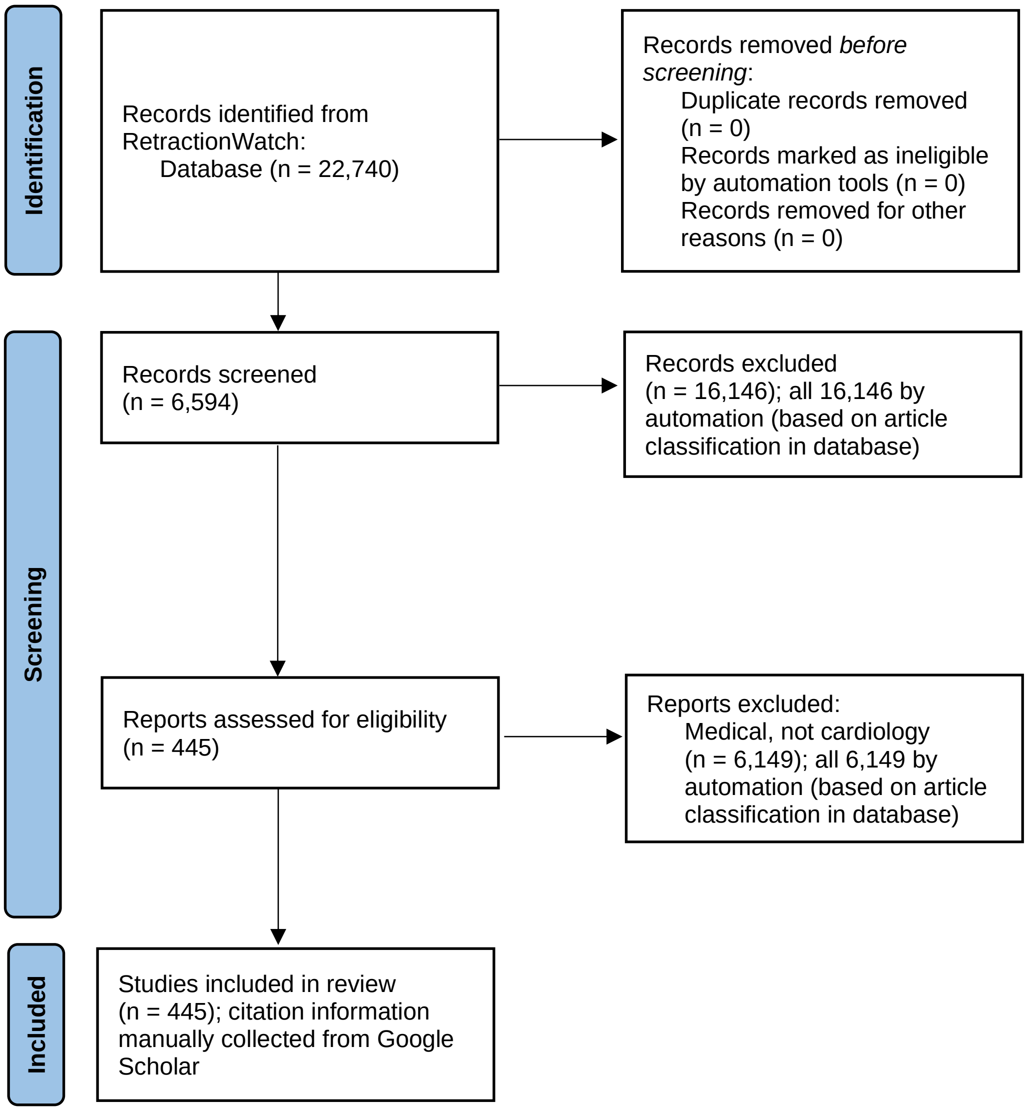

Supplemental Figure. Data flow diagram showing selection of relevant articles from Retraction Watch database and supplementation with Google Scholar DOI-matched citation data. *Template from:* Page MJ, McKenzie JE, Bossuyt PM, Boutron I, Hoffmann TC, Mulrow CD, et al. The PRISMA 2020 statement: an updated guideline for reporting systematic reviews. BMJ 2021;372:n71. doi: 10.1136/bmj.n71.

© 2021 Wadhwa RR et al. *JAMA Network Open*.
